# Cocaine-Induced Toxic Leukoencephalopathy: A Case Report

**DOI:** 10.7759/cureus.61098

**Published:** 2024-05-26

**Authors:** Zachary Ellis, Cody Stalnaker, Kelley Bellia, Odalys E Lara Garcia

**Affiliations:** 1 Internal Medicine, Baptist Memorial Hospital, Oxford, USA; 2 Internal Medicine, William Carey University College of Osteopathic Medicine, Hattiesburg, USA

**Keywords:** levamisole, drug and substance abuse, toxic leukoencephalopathy, cocaine toxicity, cocaine use

## Abstract

Cocaine is a widely abused controlled substance. Cocaine use is associated with a myriad of side effects and a sequelae of consequences secondary to its harmful nature and potential adulterants, the most recently described and less known sequelae being leukoencephalopathy. In our case, we describe a 58-year-old male who presented to the ED with agitation and acute stroke-like symptoms with reported rapid onset. Cocaine induced toxic leukoencephalopathy is a diagnosis of exclusion, thus other etiologies of disease were ruled out in a full neurological and infectious workup; most importantly consisting of extensive brain imaging, alluding to the diagnosis of acute cocaine induced toxic leukoencephalopathy in an individual with a confirmed history of cocaine and cannabinoid abuse. Although there is no targeted therapy for the condition to our knowledge, we utilized a supportive approach to treatment in contrast to other reported treatment modalities which included the use of steroids, plasma exchange, and intravenous immunoglobulin. Furthermore, we describe the clinical evaluation and treatment throughout the patient’s hospital course with his eventual marked improvement from initial presentation.

## Introduction

Leukoencephalopathy is a syndrome of neurologic deficits, including alteration of mental status caused by pathologic changes in the cerebral white matter [1]. Toxic leukoencephalopathy is a broad diagnosis that encompasses a variety of exposures and clinical presentations [1]. This disease process particularly involves white-matter tracts devoted to higher cerebral function, causing clinical features that range from inattention, forgetfulness, and changes in personality to dementia, coma, and death [2]. The syndrome is increasingly being recognized among patients in whom neurobehavioral disturbance develops after exposure to toxins [2]. Diagnosis usually requires but is not limited to a high index of suspicion, documented exposure to a toxin, neurobehavioral deficits, and neuroradiologic abnormalities [2,3].

There are many causes of toxic leukoencephalopathy. The most common etiologies of toxic leukoencephalopathy are cranial radiation therapy, chemotherapeutic agents, environmental toxins and recreational drug use [2,3]. These substances tend to be modified with certain adulterants which may contribute to the likelihood of developing leukoencephalopathy. Levamisole is becoming an increasingly more common adulterant that is used as a high-performance cutting agent and additive to cocaine. Levamisole is an antihelminthic drug approved for use in veterinary medicine in the United States. It was pulled as an immunomodulatory agent from the US market in 2000 due to severe side effects; including, but not limited to severe agranulocytosis, vasculitis, hemorrhage, and many neurological side effects including dizziness, headache, confusion, seizures, psychosis, and encephalopathy [4]. Previous animal studies suggest that levamisole potentiates nicotinic acetylcholinergic effects on the central nervous system, thus prolonging cocaine-induced euphoria by providing an amphetamine-like stimulant effect that lasts long after the direct effects of cocaine have worn off [4]. Recent reports suggest that 80% of cocaine seized in the United States is laced with the anthelmintic agent [5].

Neuroradiologic and neuropathological studies have been the primary means of identifying toxins that target cerebral white matter [2,3]. MRI findings characteristic of drugs of abuse, including cocaine, involve symmetrically increased T2 and T2-fluid attenuated inversion recovery (FLAIR) signal intensity in the cerebellar white matter and the posterior limb of the internal capsule [6]. There is also symmetrically increased T2 and T2-FLAIR signal intensity involving the supratentorial white matter as seen with other leukoencephalopathies [6].

Cocaine-induced toxic leukoencephalopathy is an uncommon manifestation of the adverse effects related to the consumption of cocaine and possible adulterants. This case report aims to shed light on the dangerous less common sequela of cocaine abuse with the intention to spread awareness and aid other healthcare providers in creating a pattern of recognition to facilitate the diagnosis of cocaine-induced toxic leukoencephalopathy.

## Case presentation

This is a case of a 58-year-old male with a past medical history of polysubstance abuse, ischemic stroke, hypertension, and type 2 diabetes mellitus who presented to the emergency department (ED) with stroke-like symptoms. One day prior to admission, the patient was noted to have motor weakness and sensory loss in his right upper extremity. The following morning, his condition worsened with the addition of a headache, behavioral changes, nausea, and vomiting. Upon presentation to the ED, the headache, nausea, and vomiting acutely worsened and the patient developed restlessness and agitation which provoked crying due to the severity of the pain. A more thorough history revealed the patient recently used cocaine. The patient's wife admitted that the patient occasionally uses cannabinoids and cocaine, however, the exact frequency, duration, and amount could not be obtained. In the ED, the patient was aphasic with generalized weakness, worse on the right side with a left gaze preference suggestive of an acute stroke in the left middle cerebral artery (MCA) territory. At this point, he continued to remain agitated and his altered state of consciousness eventually declined to become unresponsive and nonverbal. His vitals and labs were notable for a blood pressure of 232/136 and a blood glucose of 447. A urine drug screen was positive for cannabinoids and cocaine (levamisole was not tested). CT head showed scattered areas of low attenuation in the periventricular and subcortical white matter characteristic of mid-small vessel changes with no acute stroke or hemorrhage identified.

At time of admission, Neurology was consulted and a comprehensive workup was completed including MRI Brain, MRA Head/Neck, and a full work-up for stroke etiologies including transthoracic echocardiogram (TTE) with bubble study, blood cultures, EKG/telemetry, lipid panel, A1c, thyroid-stimulating hormone (TSH), folate, and B12. The initial workup was unrevealing and the MRI was still pending. The patient remained unresponsive and nonverbal, therefore, an EEG, rapid plasma reagin (RPR), Venereal Diseases Research Laboratory (VDRL), HIV, heavy metals, and lumbar puncture with meningitis and paraneoplastic panel were performed. The patient began spiking fevers which led the treatment team to consider a more infectious route of disease. Broad spectrum coverage for infectious etiologies was initiated while the infectious workup remained pending. The patient was started on treatment consisting of acyclovir, vancomycin, ampicillin-sulbactam, and cefepime.

**Figure 1:**
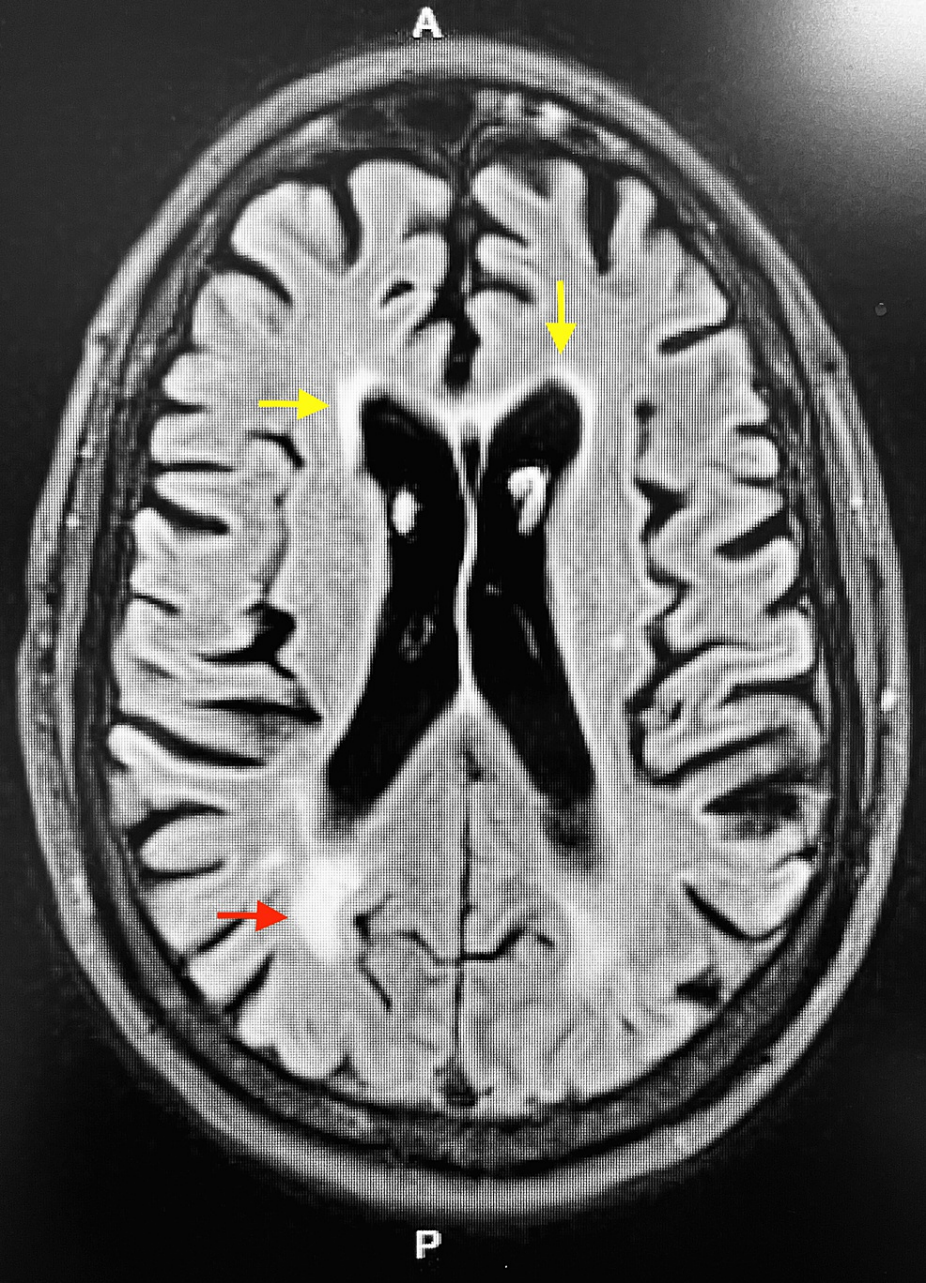
MRI Brain showing increased T2 signal within the periventricular (yellow arrows) and subcortical (red arrow) white matter; characteristic of toxic leukoencephalopathy.

**Figure 2:**
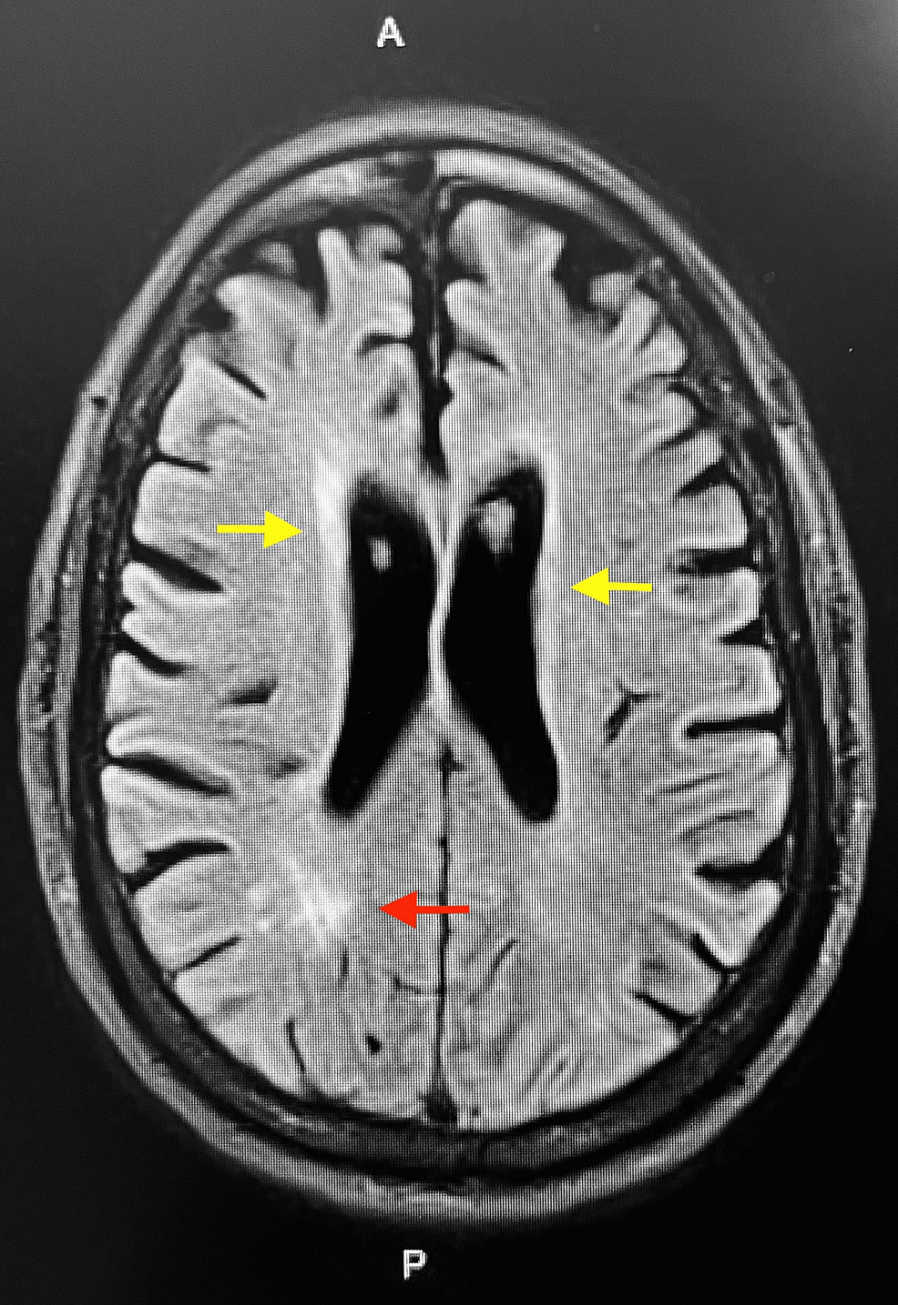
Alternative transverse sequence of MRI Brain showing increased T2 signal within the periventricular (yellow arrows) and subcortical (red arrow) white matter; characteristic of toxic leukoencephalopathy.

An MRI Brain without contrast (Figure 1) showed mild generalized cerebral atrophy with equal prominence of the ventricular system and cortical sulci. There were areas of increased T2 signal within the periventricular and subcortical white matter characteristic of mild small vessel ischemic disease (Figure 2). Old lacunar infarctions were noted in the left side of the pons, each thalamus, and the left subinsular region. The diffusion images revealed no areas of increased signal to suggest an acute stroke. EEG was consistent with mild encephalopathy of toxic, metabolic, and/or degenerative etiology. Outside of a positive urine drug screen (UDS) for cannabinoids and cocaine and an A1c of 12.9%, all other laboratory testing was unremarkable including lumbar puncture and cultures.

Despite the initiation of broad-spectrum antibiotics and antivirals, no clinical improvement was seen by day four of admission. The decision was made by our multidisciplinary team to discontinue broad-spectrum antibiotic and antiviral therapy due to low suspicion for an infectious etiology. On days five to six of admission, the patient remarkably began to recover and became more alert and oriented. Over the next several days, with supportive care, the patient returned to his baseline mental and functional status. 

## Discussion

Toxic leukoencephalopathy related to cocaine abuse is a rare complication of cocaine usage. Other more likely cocaine-related issues include its cardiovascular effects and increased stroke risk. The etiology of this condition is poorly understood. Histologic findings in patients with cocaine induced leukoencephalopathy show widespread confluent vacuolar degeneration of the deep white matter, with profound axonal loss and evidence of axonal injury in adjacent normal appearing white matter [7,8]. This attempts to suggest that the axonal injury precedes the myelin vacuolar change. However, it appears the axonal injury and vacuolar change appear to parallel each other. Interestingly, the prominent axonal involvement is unusual in other drugs of abuse, such as heroin. In heroin-induced leukoencephalopathy, the axons are typically preserved, thus further explorations are necessary to rule out other diseases with similar presentation [8]. 

Imaging remains one of the most important factors in diagnosis which is best observed with T2 FLAIR MRI with no contrast enhancement [8]. Information presented in other case reports has led to a popular hypothesis describing asymptomatic cocaine-dependent subjects have significantly more white-matter lesions on MRI than control subjects, suggesting that those who abuse cocaine are at increased risk for vasospasm-induced ischemia and infarction of white matter [3,9]. Some research may even suggest an age-related component [9].

An important contributor in cocaine-induced leukoencephalopathy is levamisole. Before being pulled from the US market due to its adverse effects, in 1991 levamisole was approved for adjuvant treatment for colorectal cancer [10]. Shortly thereafter, multifocal leukoencephalopathy had been associated with the drug as evidenced by three patients receiving therapy for colorectal cancer in 1992. Levamisole has since been linked to agranulocytosis, vasculitis, arthralgias, retiform purpura, skin necrosis, and more [4]. Recent reports suggest that 80% of cocaine seized in the United States is laced with the anthelmintic agent [5]. Improved detection methods using gas chromatography/mass spectrometry on urine samples are now being employed at some institutions [4,11]. Utilizing this method, levamisole was found in 68% of patients who tested positive for cocaine on standard urine toxicology screen [4,11]. Although this is useful, levamisole is not on a standard drug screen and there was no option to test for this substance at our facility.

There have been cases in which high dose steroids, plasmapheresis, and intravenous immunoglobulin have been utilized resulting in both favorable and unfavorable outcomes [6,12-14]. Evidence is sparse regarding the topic; however, currently there is no proven treatment for toxic leukoencephalopathy. In patients that do not improve over the course of days with supportive therapy; the use of steroids, plasmapheresis, and/or intravenous immunoglobulin may be beneficial. This approach is hypothesized to combat inflammation and edema imposed by the neurotoxic effects of cocaine; yet, it cannot be ascertained whether early removal of the offending agent and/or induction of steroids provided the most benefit in certain cases that used this method [13]. 

Although most cases show improvement and favorable recovery, there are reports of individuals with unfavorable outcomes raising the question of effectiveness of treatment. The course of disease ranges from about five days to two weeks among cases which is consistent with our patient’s presentation [6,12,13]. Similar to other reported approaches to diagnosis, the diagnostic evaluation roughly parallels each other in regard to ruling out alternative diseases. Differences arise most notably in our case compared to others in regards to management of disease. Due to insufficient evidence of efficacy and lacking guidelines for treatment, we found no indication for the use of steroids, plasmapheresis or intravenous immunoglobulin. After an infectious etiology of disease was ruled out and broad-spectrum antibiotic/antiviral agents were discontinued, a conservative approach to treatment with supportive therapy was pursued in our case which resulted in a favorable outcome. Additionally, although recovery time may vary between cases due to the extent of damage, we achieved the same favorable outcome more expeditiously than other reported cases.

## Conclusions

Due to the lack of information regarding the topic, cocaine-induced toxic leukoencephalopathy remains an understudied topic and may remain misdiagnosed in patients. Multiple theories have been postulated through other similar presentations to understand the pathophysiology of the disease and disease presentation to further aid in developing treatment for the condition. Additionally, the results of treatment have varied from case to case. This case of cocaine-induced toxic leukoencephalopathy demonstrates a unique presentation due to its conservative approach to treatment through the use of supportive therapy alone. Although some clinicians have previously utilized intravenous immunoglobulins, plasma exchange, and intravenous steroids to combat the inflammatory aspect of the disease, in our case, a broad-spectrum antibiotic/antiviral regimen was initiated with eventual discontinuation resulting in a favorable outcome. No treatment guidelines or comparative studies for cocaine-induced leukoencephalopathy exist to our knowledge, thus we cannot suggest that supportive measures should be taken over plasmapheresis, steroids, or intravenous immunoglobulin as both have demonstrated favorable outcomes in previous cases; however, from our case it can be concluded that supportive therapy may be equally efficacious as other reported treatment regimens. Further research and guidance are needed to assist clinicians in the diagnosis and treatment of cocaine-induced toxic leukoencephalopathy.
